# History of facial pain diagnosis

**DOI:** 10.1177/0333102417691045

**Published:** 2017-02-09

**Authors:** Joanna M Zakrzewska, Troels S Jensen

**Affiliations:** 1 UCLH NHS Foundation Trust, London, UK; 2 IDNC and DPRC Department of Neurology, Aarhus University Hospital, Aarhus, Denmark

**Keywords:** History, trigeminal neuralgia, trigeminal autonomic cephalagias, persistent idiopathic facial pain, temporomandibular disorders

## Abstract

**Premise:**

Facial pain refers to a heterogeneous group of clinically and etiologically different conditions with the common clinical feature of pain in the facial area. Among these conditions, trigeminal neuralgia (TN), persistent idiopathic facial pain, temporomandibular joint pain, and trigeminal autonomic cephalalgias (TAC) are the most well described conditions.

**Conclusion:**

TN has been known for centuries, and is recognised by its characteristic and almost pathognomonic clinical features. The other facial pain conditions are less well defined, and over the years there has been confusion about their classification.

## Introduction

Facial pain is pain localised to the face, and the diagnosis of facial pains has puzzled clinicians for centuries. Some of the confusion is related to the delimitation of the facial structure and how pain is classified. The face is here defined as the part of the head that is limited by the hairline, by the front attachment of the ear and by the lower jaw, both the rear edge and the lower horizontal part of the jaw. The face also includes the oral and nasal cavity, the sinuses, the orbital cavity and the temporomandibular joint. Pain in the facial region can be classified in multiple ways, for example according to underlying pathology (malignant vs. non-malignant), the temporal course (acute vs. chronic), underlying pathophysiology (neuropathic, inflammatory or idiopathic), localisation (superficial vs. deep), the specific structure involved (the sinus joint, skin etc), and underlying etiology (infection, tumour etc). In some instances, the diagnosis of facial pain focuses on the involved structure, for example temporomandibular joint disorder, in other cases it is the underlying pathology (sinusitis), and in others it is the specific character of the pain that will dictate the diagnosis (e.g. trigeminal neuralgia).

Facial pains, like other pains, are purely subjective and for some painful conditions there are no obvious objective signs, so the diagnosis very much relies on the descriptive characteristics of the pain. We will limit the discussion in this paper to four major types of facial pains: Trigeminal neuralgia, trigeminal autonomic cephalalgias (TACs), persistent idiopathic facial pain (atypical facial pain), and temporomandibular disorders and summarise a few historical aspects of diagnosing these conditions.

## Trigeminal Neuralgia (TN)

The history of TN has been covered in detail in Stookey and Ransohoff’s book (1) on this topic, so only various key features will be highlighted. The first detailed description of TN was provided by John Locke. In a letter dated 4 December 1677, he gave a comprehensive description of TN based on an attack he had witnessed. It described a case of TN in the Countess of Northumberland, and John Locke here made it clear that the pain was unilateral, involving not only the face but also the mouth and tongue (2). It was described as episodic and triggered by talking. Locke reported contortion of her face during an attack. This latter aspect was also noted by André (3) in 1756, who called trigeminal neuralgia “tic douloureux” because the facial pain was associated with transient twitching of the facial muscles.

**Figure 1.**
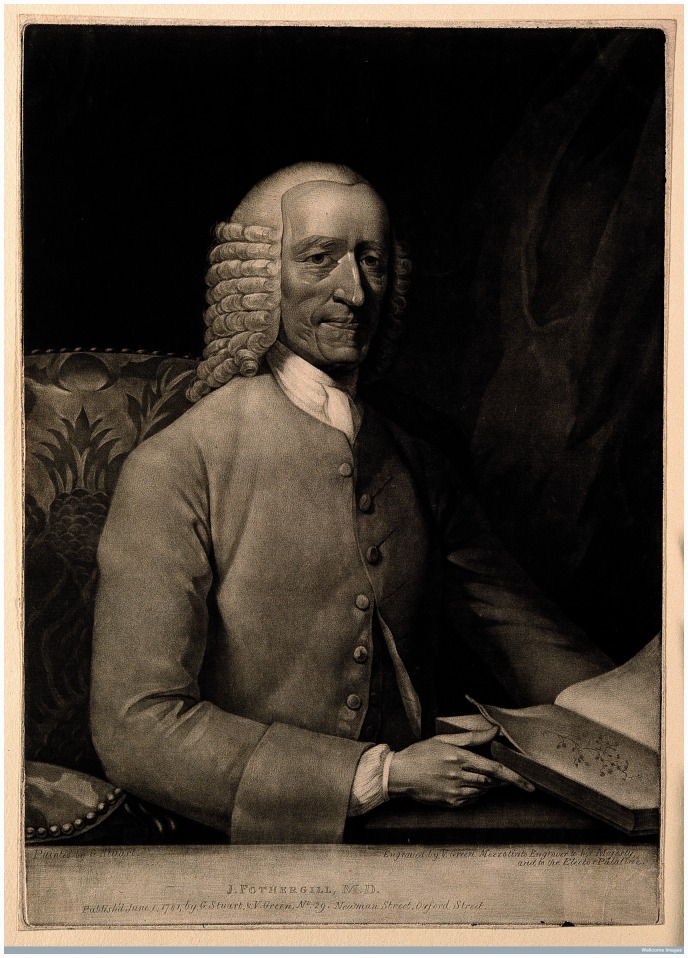
John Fothergill, 1712–1780. Courtesy Wellcome Images.

In 1773, Fothergill (Figure 1) (4) gave a detailed description of 14 cases in his paper “*On a Painful Affliction of the Face”*, and termed it Fothergill’s disease. “*The affection seems to be peculiar to persons advancing in years, and to women more than to men … . The pain comes suddenly and is excruciating; it lasts but a short time, perhaps a quarter or half a minute, and then goes off; it returns at irregular intervals, sometimes in half an hour, sometimes there are two or three repetitions in a few minutes … .Eating will bring it on some persons. Talking, or the least motion of the muscles of the face affects others; the gentlest touch of a hand or a handkerchief will sometimes bring on the pain, whilst a strong pressure on the part has no effect*.” This description encapsulates many of the key features of the condition: The abrupt nature, the excruciating character of the pain, the short lasting duration of attacks and the presence of trigger factors. Harris (5) noted that the pain is provoked by light touch but also mentioned emotional factors, cold wind, and trauma to the face as precipitating factors. Kugelberg and Lindblom (6) highlighted that thermal stimuli are not triggers but that touch, even a single displaced hair, can provoke an attack.

### Duration of pain attacks

The current diagnostic criteria state that attacks last from a few seconds to two minutes. But as has been noted in Andre’s (3) classical description, the periodic pain became so frequent that she “ *rarely had five or six minutes of peace in an hour*”. Krause (7) in 1913 pointed out that in later stages the attacks begin to last longer and longer, “*I have repeatedly seen that the pain free intervals in their duration, completely recede in comparison to the terrible attacks, so much so that strictly speaking one is hardly warranted in speaking of attacks*”. Henderson (8) highlights that the pain lasts for seconds but then goes on to say that “*a series of rapidly recurring spasms may be described as continuous pain lasting an hour or longer*”. Rasmussen (9) in his dissertation also emphasised the short duration of attacks (less than two minutes). He also presented graphic illustrations of trigeminal neuralgia in patients, demonstrating both the duration of individual attacks, the duration of series of attacks and length of pain free intervals. It was suggested that drawings might be used as a tool to distinguish TN from atypical facial pain. This could support the idea that patients may have different types of pains, ranging from single attacks to a series of prolonged attacks, and that these can vary over time. A refractory period i.e., the inability to elicit an attack immediately after another attack, was described in 1959 by Kugelberg and Lindblom (6) and was considered to be a distinguishing feature of TN.

#### Location of pain and trigger zones

The right side of the face has been reported to be more commonly affected than the left side, and Harris (5) attributed the excess of right-sided cases to the fact that oral hygiene in right-handed patients was worse on the right side. Henderson (8) pointed out that triggers could precipitate pain in another division and he described two trigger zones: One around the mouth and one at the lower end of the nose. This is in agreement with Kugelburg and Lindblom (6), who pointed out similar trigger areas. Stookey and Ransohoff (1) attribute the description of trigger areas to Patrick, writing in 1914 “*sometimes these zones are particularly restricted: A spot no larger than a finger nail*” and Kugelberg and Lindblom (6) observed that the area can be as small as 1–2 mm^2^, but in others trigger areas can be diffuse and large.

#### Etiology

The etiology of TN was unknown for a long period of time. Interestingly, Shoja (10) et al. has recently translated the writings of a mediaeval Persian physician, Esmail Jorjani. In his book, called Treasure of the Khawarazm Shah (1042–1137), they found not only a description of TN but also a suggestion that a nerve vessel conflict may be present. Jorjani writes: *“If a patient with toothache complains of sudden onset jaw pain with the sense of muscle convulsion and anxiety, you must know that the pathology is at the nerves to the teeth and that the cause of muscle convulsion and anxiety is the artery moving close to the nerves or in contact with them”.* The idea of vascular compression was pointed out first by Dandy (11) in the 1920s. Following his observation, decompression was undertaken by others, and Gardner et al. (12) suggested that compression of the sensory root might be one of the mechanisms that would elicit the attacks of TN. However, it was not until Jannetta (13) in 1977, using a microscope, reported his result from microvascular decompression that the idea of vascular compression of the trigeminal root as an etiological factor was widely accepted. Within the last 30–40 years, it has become evident that in a large group of patients with TN there is compression of the trigeminal nerve root near the dorsal root entry zone by a blood vessel, and that this compression is causally linked to TN. It has also become clear that the mere contact of the vessel to nerve root is not sufficient and that compression, atrophic thinning of the nerve or distortion are additional requirements for postulating a causal relationship between vasculature and TN.

There are other causes of TN. Wartenberg (14) had previously pointed out that several cases can be associated with tumours, yet all have classical features of TN. Subsequently, it has been noted that posterior fossa meningiomas or neuromas are found in about 2% of cases presenting with a typical TN (15). When tumours are located more peripherally, they are likely to be associated with sensory disturbances (15). Multiple sclerosis is associated with TN and is seen in 2–4% of patients with MS (16). It can even be the first sign of MS (17), and some of these patients have bilateral pain.

In 1988, the first classification and diagnostic criteria for headache disorders, cranial neuralgias and facial pain was published by the International Headache Society (18). This classification has subsequently been revised (19). In the third beta version (20) from 2013, TN is now divided into three classes: Classical TN with morphological changes in the trigeminal root by a vessel, secondary TN, where the neuralgia is secondary to an identifiable neurological disease, and idiopathic TN where the etiology is unknown.

### Trigeminal autonomic cephalalgias

The trigeminal autonomic cephalalgias (TACs) are a group of headache disorders that are characterised by unilateral pain within the trigeminal innervation territory and are associated with ipsilateral autonomic symptoms and signs. These conditions include cluster headache, paroxysmal hemicrania, and short-lasting unilateral neuralgiform headache with conjunctival injection and tearing (SUNCT). These conditions are all characterised by short-lasting pain, some localised in the facial region and some in the head, and accompanied by different autonomic features.

#### Cluster headache

The best known TAC condition is cluster headache. The first descriptions are variously accredited to van Swieten in 1745 (21), Tulp in 1641 (22), and Harris in 1926 (5), but it was Horton who provided the first description of what was called Horton’s histamine headache. Horton (23) described a specific form characterised by headache, facial pain, tearing and running nose and swelling around the eye that could be induced by subcutaneous injection of histamine. A wide variety of names were subsequently used, probably for the same diagnosis, which included among others “ciliary neuralgia”, “erythromelalgia of the head,” “Sluder’s neuralgia,” “sphenopalatine neuralgia,” and “migrainous neuralgia”, but it was Kunkle et al. (24) in 1952 who referred to it as a cluster headache.

#### SUNCT/SUNA

The first cases of SUNCT were described by Sjaastad in the late 1970s in a local journal, and three cases are described in 1989 by Sjaastad et al. (25). Another condition termed SUNA, short neuralgiform headache with autonomic features, has been added as sometimes autonomic features other than conjunctival injection and tearing are noted (25). Interestingly, in 1903 Moore (27) already wrote when describing the features of TN that *“it may cause the eyes to water and the nose to discharge as well as result in redness in “the track” of the nerve”*.

#### Paroxysmal hemicrania

The fourth condition in this group is paroxysmal hemicrania, described as a chronic paroxysmal hemicrania responsive to indomethacin by Sjaastad and Dale (28) in 1974. The condition of hemicrania continua was later described by Sjaastad and Spierings (29) in 1984, in which the headache is continuous.

## Persistent idiopathic facial pain (PIFP), (atypical facial pain, chronic idiopathic facial pain), atypical odontalgia (AO)

Persistent Idiopathic Facial Pain (PFIP) is also known as atypical facial pain or non-neuralgiform facial pain. Atypical facial pain (AFP) was first described in 1924 by Frazier and Russell (30), who noted that 10–15% of patients with chronic facial pain had pain that differed in nature from trigeminal neuralgia. They termed these conditions “atypical neuralgia”, as they did not have the characteristic clinical pattern of neuralgia. A more accurate name for this condition would, according to Rasmussen (9), be non-neuralgiform facial pain. It is often used as a diagnosis of exclusion.

The pain appears to present more commonly in women, and was originally described as a psychiatric condition often in young professional women in whom hysterical, obsessional and anxiety traits had been detected. Further details of the current diagnostic criteria are to be found in a subsequent paper in this series.

Atypical odontalgia was originally described as “idiopathic periodontalgia” by Harris (31). It has further been described by Baad-Hansen (32) and List (33), and they provide full descriptions and show that somatosensory abnormalities can be present. More recently, the term “persistent dentoalveolar pain” has been suggested (34).

### Temporomandibular disorders (TMD)

Costen (35) is credited with describing this condition first as “a syndrome of ear and sinus symptoms dependent upon disturbed function of the joint”. In his 1934 description, he listed 14 symptoms and for some time the term “Costen syndrome” was used. However, in 1959, Schwartz (36) named it temporomandibular joint pain dysfunction, whereas Laskin (37) called it myofascial pain dysfunction as opinion was divided as to the source of the pain: muscles or joint. Over the years, varying classifications have been used, by the International Association for the Study of Pain in 1986 and by the International Headache Society (18–20). In 1992, Dworkin and le Resche (38) proposed the Research Diagnostic Criteria (RDC/TMD), which provided a dual diagnostic system – physical presentation and psychological factors – that were accepted by the orofacial community and were further refined in 2014 (39). A distinction is made between conditions affecting the muscles of mastication only compared to those that also involve the joint either exclusively or in combination with the muscles. The psychological aspects are ascertained through a series of psychometrically validated questionnaires such as the Chronic Graded Pain Scale and the Multidimensional Pain Inventory (MPI). Other features of TMD are discussed in other papers in this series.

## Clinical implications

- Trigeminal neuralgia has been recognised for centuries. Former studies have pointed out the classical features, with unilateral attacks of shock-like pains in the facial region, provoked by trivial stimuli and with remissions.
- Nomenclature of other chronic facial pains is confusing and variable.
- Temporomandibular disorders have recently been well described and include psychological features.
